# Preliminary outcomes of five-year survival for ovarian malignancies in profiled Serbian Oncology Centre

**DOI:** 10.1016/j.clinsp.2023.100204

**Published:** 2023-05-04

**Authors:** Bojana Gutic, Tatjana Bozanovic, Aljosa Mandic, Stefan Dugalic, Jovana Todorovic, Miroslava Gojnic Dugalic, Demet Sengul, Dzenana A. Detanac, Ilker Sengul, Dzemail Detanac, Tugrul Kesicioglu, José Maria Soares Junior

**Affiliations:** a Oncology Institute of Vojvodina, Sremska Kamenica, Faculty of Medicine University of Novi Sad, Faculty of Medicine University of Belgrade, Belgrade, Serbia; b Department of Gynecology and Obstetrics, University Clinical Center of Serbia, Clinic for Gynecology and Obstetrics, Faculty of Medicine University of Belgrade, Belgrade, Serbia; c Institute for Social Medicine, University of Belgrade, Belgrade, Serbia; d Department of Pathology, Faculty of Medicine, Giresun University, Giresun, Turkey; e Department of Ophthalmology, General Hospital Novi Pazar, Novi Pazar, Serbia; f Division of Endocrine Surgery, Faculty of Medicine, Giresun University, Giresun, Turkey; g Department of General Surgery, Faculty of Medicine, Giresun University, Giresun, Turkey; h Department of General Surgery, General Hospital Novi Pazar, Novi Pazar, Serbia; i Disciplina de Ginecologia, Departamento de Obstetrícia e Ginecologia, Hospital das Clínicas, Faculdade de Medicina da Universidade de São Paulo, São Paulo, SP, Brasil

**Keywords:** Surgery, Ovary, Carcinoma, Oncology, Histopathology, Pathology

## Abstract

**Objective:**

The present study purposed to determine characteristics of ovarian carcinoma and to analyze predictors of survival in patients with ovarian carcinoma.

**Method:**

A retrospective cohort study was conducted including the patients with diagnosed ovarian carcinoma treated at the Clinic for Operative Oncology, Oncology Institute of Vojvodina in the period from January 2012 to December 2016. Seventy-two women with ovarian carcinoma were included in the analysis. The data about the histological type of tumor, disease stage, treatment, lymphatic infiltration, and surgical procedure were collected retrospectively, using the database of the institution where the research was conducted (BirPis 21 SRC Infonet DOO ‒ Information System Oncology Institute of Vojvodina). Descriptive statistics and multivariate analysis using Cox proportional hazards model were performed.

**Results:**

The univariate Cox regression analysis identified histology, tumor grade, FIGO (International Federation of Gynecology and Obstetrics) stage, NACT (Neoadjuvant Chemotherapy), number of therapy cycles, type of surgery, and chemotherapy response as independent predictors of mortality. Finally, the type of tumor and chemotherapy response had an increased hazard ratio for mortality in the multivariate Cox regression model. Herewith, the percentage of high-grade, advanced-stage ovarian cancer patients with complete response to chemotherapy, absence of recurrent disease, and lymphovascular space invasion were significant predictors of survival in patients with ovarian carcinoma.

**Conclusions:**

Herein, emerging data regarding precision medicine and molecular-based personalized treatments are promising and will likely modify the way the authors provide multiple lines of treatments in the near future.

## Highlights

• Analyzing predictors of survival in patients with ovarian carcinoma is crucial.
• Independent estimators of mortality in ovarian carcinoma, are more precise by identifying histopathologic tumor grade, FIGO, and NACT.
• The aforementioned predictors also involve the number of therapeutic cycles, type of surgery, and chemotherapy response.
• Poor chemotherapy response increases the hazard ratio for mortality.
• Significant predictors of survival in ovarian carcinoma are the absence of recurrent disease and lymphovascular space invasion.

## Introduction

With the aging of the world's population, the authors are faced with changes in morbidity and mortality causes and cancer is becoming the leading cause of death. It is estimated that in the continent of Europe, which counts close to 10% of the world's population, there are about 23.4% newly diagnosed cancers and about 20.3% cancer-related deaths. In the world population, lung cancer is the most common cancer type and the most common cause of death in cancer cases. Breast cancer is the most frequently diagnosed and the most common cause of cancer-related death among women [1].

Although ovarian cancer, *per se*, is not very common and accounts for only 3% of the general population of women, it is the fifth most common cause of death among women diagnosed with malignancy [2]. Age-adjusted incidence is calculated as 12.5 per 100.000 women [3]. The incidence and mortality rates are higher in older women and increase with age, hence the probability of getting this malignancy is higher in the n who are 50 years. However, the disease can be diagnosed at any age [4]. Genetics is a significant risk factor for ovarian cancer, and there is also hereditary breast and ovarian cancer syndrome, which occurs in one in 500 women. That is the result of an autosomal dominant mutation of the BRCA1 or BRCA2 gene. Mutation of this specific gene (one or two) increases the risk of ovarian cancer, breast cancer, and other malignancy and it is considered responsible for 23 to even a 54-lifetime risk of ovarian cancer [5], [6], [7], [8]. Often and repeated ovulatory rupture, repair, and partial scarification may lead to the mutation of the gene in the ovary itself and increase the risk of ovarian cancer which can also explain the protective role of oral contraceptives, late menarche, early menopause, multiparity, and breastfeeding [6,9]. Besides this factor, which cannot be changed or modified, several risk factors are shown to be risk factors for ovarian carcinoma. These are obesity, smoking, a high-starch, and high-fat diet, and a sedentary lifestyle. However, they are not proven as the primary cause of this malignancy. On the other hand, fiber intake, carotene, vitamin C and E use, unsaturated fatty intake, and physical activity are identified as protective factors [10].

Ovarian cancer, as mentioned above has a high mortality rate and is the most common death from gynecological tumors.[11]. Early diagnosis is rare and symptoms are not specific, so only 15% of tumor cases are diagnosed in the early stage (FIGO classification stage I) [12]. The majority of ovarian cancer patients are diagnosed in the advanced stage of the disease, which is associated with poor prognosis compared to localized disease where the 5-year survival is about 92% [13]. In the case of the advanced tumor stage, the 5-year survival rate is low and is only about 29.2% [14]. In addition, 70%‒90% of women with ovarian cancer in the late stage experience the recurrence of the disease within 18 months of diagnosis [15].

Several studies have focused on the immunology, pathogenesis, and therapy of ovarian cancer, suggesting the role of T-cell infiltration and Tumor-Associated Macrophage (TAM) expression on both, ovarian cancer cells *in vitro* and *in vivo* [16]. PD1 (Programmed Cell Death 1) with its role in cell apoptosis and PD1/ PD-L1 (Programmed Cell Death 1 Ligand) complex are the important immune checkpoint in the proliferation and development of tumors [17]. Tumor cells with PD-L1 expressed on their surface bind to the PD-1 receptor of T-lymphocytes and inactivate the immune response of the host [18]. Two recent meta-analyses suggested that PD-L1 expression was not linked to tumor histology, Overall Survival (OS), and Progression-Free Survival (PFS), but that PD-L1 mRNA expression was closely correlated with poor PFS [19]. The study focused on morphology and molecular genetics gives a new concept to ovarian carcinoma, taking into consideration differences in pathogenesis, clinical presentation, nature, and disease course, as well as the prognosis. These differences make a clear distinction between two types of ovarian cancer type I and II. Type II is more aggressive, contrary to type I, and possesses an advanced disease stage at the moment of diagnosis [16], [17], [18], [19], [20], [21], [22].

Despite poor prognosis in women with ovarian cancers, outcomes may be very heterogenous and differ from each other, with approximately a third of women achieving long-term survival (more than 9 years) [23]. These long-term survivors also include a proportion of women with poor clinical characteristics at diagnosis, such as advanced-stage disease or performing suboptimal debulking surgery. The predictors of long-term survival are not well understood, and it remains unknown whether associations between patient characteristics and risk of mortality differ across the survival trajectory and whether these associations vary according to histologic type [24]. The present study purposed to determine the characteristics of ovarian carcinoma and to analyze predictors of survival in patients with ovarian carcinoma.

## Material and methods

This study was conducted according to the declaration of Helsinki and approved by the Ethical Board of Oncology Institute of Vojvodina under the 4/20/2-3707/2-6 approval number. It has been designed as a retrospective cohort study and included patients with diagnosed ovarian carcinoma treated at the Clinic of Operative Oncology, Oncology Institute of Vojvodina in the period from 2012 to 2016. Clinical and radiological assessment was performed during the five-year follow-up period. Of 75 patients with ovarian carcinoma 72 were included in the analysis. Three of 75 cases were excluded due to missing follow-up. The data about the histological type of tumor, disease stage (FIGO classification 2018), treatment, Lymphovascular Space Invasion (LVSI), type of surgery, and follow-up had been collected retrospectively from the database (BirPis 21 SRC Infonet DOO ‒ Information System Oncology Institute of Vojvodina) of the institution where the research was conducted. The mentioned characteristics were analyzed as survival predictors of ovarian cancer. The progression-free survival and overall survival were used.

For this purpose, the present study analyzed women with histopathologically confirmed ovarian cancer. The tumor tissue was obtained during a biopsy or surgical procedure. Women older than 18 years of age at the moment of diagnosis, with histopathological confirmation of ovarian cancer, FIGO stages I, II, III , and IV had been included and divided into low- and high-grade tumor groups. The type of surgery was defined as complete debulking with no residual disease, optimal with residual disease less than 10 mm, and suboptimal with a residual disease with more than 10 mm. Those with tumors of other localization and missing follow-up were excluded from the analysis.

### Statistical analysis

Descriptive statistics were calculated for the baseline patients’ characteristics and outcome measures. The baseline differences between groups were analyzed using the Mann-Whitney *U* test for continuous variables, and the Pearson Chi-Squared test for categorical variables. The survival curves were estimated using the Kaplan-Meier method. The log-rank test evaluated the impact of analyzed parameters on Overall Survival (OS). The multivariate analysis using the Cox proportional hazards model was performed to determine the independent prognostic factors influencing OS which was calculated from the date of diagnosis until death or last follow-up. All tests were two-tailed and *p* < 0.05 was considered statistically significant. The IBM SPSS 21 (Chicago, IL, 2012) package was used for these analyses for the statistical evaluation.

## Results

Seventy-five patients with ovarian carcinoma were detected in the analyzed period and 72 were included in the analysis. Three of 75 patients were excluded due to missing follow-up. The baseline characteristics of the patients in this cohort were summarized in Table 1. The median age at diagnosis was 59.00 years. A larger number of women were non-survivors, although the difference was not statistically significant (*p* = 0.081) in terms of median age. However, the FIGO stage had a significant impact on survival so women who were in FIGO I and II stage had a higher chance to survive compared with those in stages III and IV (*p* < 0.001). The histopathologic type of tumor did not have a significant impact on survival (*p* = 0.081), while tumor grade had. High-grade tumors significantly decreased the chance of surviving (*p* < 0.001). The LVSI also differed between survivors and non-survivors (*p* = 0.009). The difference in NACT status was significant (*p* = 0.002) where non-survivors received NACT in 36.6% compared to survivors who received NACT in only 3.7%. A similar difference was observed in the number of cycle lines (*p* = 0.001) where the range was between 0 and 16 in non-survivors, whereas between 0 and 6 in survivors. Slightly, but still, a significant difference was recognized between survivors and non-survivors in terms of surgery type (*p* = 0.047), where non-survivors had complete surgery in the majority of cases (48.5%), while in survivors the procedure was mostly optimal (57.7%). Chemotherapy response was different between the two groups (*p* = 0.039), so non-survivors had a complete response to chemotherapy in many cases (82.1%), whereas all survivors (100%) completely responded to the therapy.

**Table 1:** Baseline characteristics.

Characteristic	All patients (*n* = 72)	Survivors (*n* = 31)	Nonsurvivors (*n* = 41)	*p*
Age, years				0.081
Median	59.00	56.00	62.00	
Range	20.00‒82.00	20.00‒82.00	39.00‒75.00	
FIGO stage				<0.001
I and II	13 (19.7%)	11 (42.3%)	2 (5.0%)	
III and IV	53 (80.3%)	15 (57.7%)	38 (95.0%)	
Tumor histology				0.081
Type I	41 (68.3%)	12 (54.5%)	29 (76.3%)	
Type II	19 (31.7%)	10 (45.5%)	9 (23.7%)	
Tumor grade				<0.001
Low grade	21 (30%)	16 (53.3%)	5 (12.5%)	
High grade	49 (70%)	14 (46.7%)	35 (87.5%)	
LVI				0.009
No	6 (14.3%)	5 (33.3%)	1 (3.7%)	
Yes	36 (85.7%)	10 (66.7%)	26 (96.3%)	
NACT Chemotherapy Response				0.002
No	52 (75.5%)	26 (96.4%)	26 (63.4%)	
Yes	16 (23.5%)	1 (3.7%)	15 (36.6%)	
Number of cycles	0.00	0.00	0.00	0.001
Lines	0.00‒16.00	0.00‒6.00	0.00‒16.00	
Type of surgery				0.047
Optimal	25 (42.4%)	15 (57.7%)	10 (30.3%)	
Suboptimal	13 (22.0%)	6 (23.1%)	7 (21.2%)	
Complete	21 (35.6%)	5 (19.2%)	16 (48.5%)	
Chemotherapy response				0.039
Complete	53 (88.3%)	21 (100%)	32 (82.1%)	
Partial response or Stable disease	7 (11.7%)	0 (0%)	7 (17.9%)	

Median overall survival was 57.00 ± 10.43 months (95% CI 36.56‒77.44). Median progression-free survival was 22.00 ± 3.60 months (95% CI 14.95–29.05) with an expected 5-year survival of 40% among the patients in the studied group. Survival analyses showed the prognostic influence of tumor type where low-grade tumors had higher OS compared to high-grade tumors (Log rank = 12.559; *p* < 0.001). The OS of cases according to LVSI status (Log rank = 4.643; *p* = 0.031) and the FIGO stage (Log rank = 9.641; *p* = 0.002) was estimated. It is shown that patients with positive LVI status had lower OS compared to those with negative LVSI status. Also, the FIGO stage was a significant predictor of OS in the studied group, meaning that patients in stage I or II had significantly better OS compared to those with FIGO stages III and IV. Survival analyses exhibited the prognostic influence of NACT on OS (Log rank = 19.300; *p* < 0.01). A statistical significance in the survival between patients according to the type of surgery (complete, optimal, and suboptimal) (Log rank = 7.178; *p* = 0.028) had been recognized and the difference in survival was observed among the patients according to chemotherapy response (Log rank = 27.475; *p* < 0.001). The presence of recurrence (Log rank = 20.060; *p* < 0.000) had also a negative impact on OS. The univariate Cox regression analysis identified histopathology, tumor grade, FIGO, NACT, number of therapy cycles, type of surgery, and chemotherapy response as independent predictors of mortality. Finally, the type of tumor and chemotherapy response had an increased hazard ratio for mortality in the multivariate Cox regression model (Table 2).

**Table 2:** Identification of risk factors for death: a multivariate analysis.

				95% CI	
	B	p	HR	Lower	Upper
Histopathology	-0.862	0.143	0.422	0.133	1.340
Tumor grade	2.460	0.009	11.702	1.861	73.587
FIGO	-0.331	0.689	0.718	0.142	3.641
NACT	1.128	0.435	3.089	0.182	52.545
Number of chemotherapy cycles	-0.101	0.800	0.904	0.416	1.968
Type of surgery	0.510	0.099	1.665	0.908	3.051
Chemotherapy response					
Regression or stable disease	1.609	0.043	4.998	1.053	23.728
Progression	1.243	0.046	3.467	1.022	11.765

## Discussion

The preliminary results of the present study exhibited an expected 5-year survival of 40%. The patients with LVSI had significantly lower survival and poor prognosis compared with those without LVSI. This is comparable with data from the literature. Li and colleagues [26]. conducted a meta-analysis that also revealed an increased risk of non-survival in patients with LVSI. The authors calculated pooled HR for all 13 articles included in the analysis and demonstrated a significantly augmented risk of disease progression in patients with LVSI presence (HR = 2.29; 95% CI 1.55‒3.37; PHR < 0.001). The subgroup analyses stratified by region and histology confirmed that LVSI presence was associated with an increased risk of disease progression in all the subgroups except the subgroup designated “Europe”. In addition, HRs for OS were available in 11 studies. The estimated pooled HR for all the studies suggested a significantly increased risk of death in the cases with LVSI presence (HR = 1.71; 95% CI 1.42‒2.07, PHR < 0.001). Subgroup analyses stratified by region and histopathology confirmed that LVSI presence was associated with an increased risk of death in all the subgroups. Even patients at an early stage had a relatively lower incidence of LVSI presence, LVSI presence was still associated with shorter progression-free survival (HR = 2.20; 95% CI 1.50‒3.21; PHR < 0.001; fixed effects model) and OS (HR = 2.76; 95% CI 1.27–6.00; PHR = 0.011). Of note, the present analysis revealed that patients with advanced stages (III and IV) had poor prognoses and survival outcomes compared to patients with early stages (I or II) which followed the results of Li et al., [25] although it included a higher number (192) of participants. The advanced stage was significantly associated with an increased risk of LVSI presence, and the univariate analysis also revealed the expression of SNAI1 and SNAI2 were all positively correlated with LVSI presence. The advanced stage (OR = 4.44; 95% CI 1.443‒13.75; *p* = 0.01) remained significantly correlated with LVSI presence [25]. According to the National Cancer Institute's Surveillance, Epidemiology, and End Results registries, > 60% of patients with Epithelial Ovarian Cancer (EOC) are diagnosed with FIGO stage III‒IV. In this setting, primary cytoreductive surgery followed by taxane- and platinum-based combination chemotherapy is a well-established management strategy. The goal of surgery should be the complete removal of all macroscopic diseases, as “complete cytoreduction” is one of the most important prognostic factors of survival. This is in accordance with the present analysis which also demonstrated a significant impact of surgery type on survival. In the analysis, there were more than 50% in the survivals group with at least optimal debulking surgery. Also, patients with stage IV disease who underwent Neoadjuvant Chemotherapy (NACT) showed significantly better PFS (median, 10.6 vs. 9.7 months; HR = 0.77; 95% CI 0.59‒1.00; P¼ = 0.049) and OS (median, 24.3 vs. 21.2 months; HR = 0.76; 95% CI 0.58‒1.00; P¼ = 0.048) than those who underwent upfront cytoreductive surgery (CRS). Of note, the present analysis did not include a comparison between surgery type and NACT but did show that response to treatment and histopathologic type of tumor were significant factors of cumulative survival [26].

For patients with recurrent ovarian cancer, the value of additional cytotoxic therapy beyond third-line chemotherapy versus halting therapy and switching to supportive care remains largely unexplored, although supportive therapy remains the only option when all other therapeutical approaches did not show any result. Kessous and colleagues [27] assessed the value of different clinical variables in predicting response to future lines of chemotherapy among 238 ovarian cancer cases. The number of previous lines of the therapy and time interval from the previous line of chemotherapy were the only clinical variables correlated with patient outcome and response to subsequent therapy. The time interval between previous and current lines of therapy was a predictor of the beneficial effect of an additional line of treatment. The median age at diagnosis was 63 years (IQR 54‒73 years), while the median BMI was 25.0 kg/m^2^ (IQR 22.0‒29.7 kg/m^2^), with 51 (21.4%) of patients were obese (BMI ≥ 30.0 kg/m^2^). The majority of cases had tumors with serous histopathology (91.6%) and FIGO Stage III cancer (82.3%) and BRCA mutations were present in 22 cases (9.2%). The median CA-125 at diagnosis was 457 U/mL (IQR 134‒1367 U/mL). Debulking surgery was conducted in 94.1% of the cases. One hundred twenty-one (50.8%) patients did not have residual disease after surgery, while 60 (25.2%) and 34 (14.3%) had a < 1 cm or ≥ 1 cm residual disease, respectively. Approximately half (53.4%) of the patients received NACT. Most patients (93.3%) received carboplatin with paclitaxel as the first chemotherapy. As expected, the majority of patients (92.3%) responded to first-line chemotherapy (PR/CR). However, response rates dropped markedly with each additional line of treatment. By line 5, over 60% of patients had PD, while only 4.3% and 12.8% had a recurrence or progressive disease, respectively. The present analysis exhibited that disease recurrence significantly impacted survival in patients with ovarian carcinoma. In addition, response to chemotherapy was a significant predictor of survival in the present study. This is to the results of the above-mentioned work of Kessous et al. [27] where the response to chemotherapy predicted OS regardless of the line of chemotherapy and the difference was most pronounced in the first line, where patients with recurrent carcinoma did not have significantly improved overall survival compared with those with progressive disease. Even some of the baseline characteristics were similar to the analysis, which further supports the authors’ work and results, even though the sample was smaller.

## Conclusion

In conclusion, as expected, the percentage of high-grade, advanced-stage ovarian cancer patients, *per se*, possessing a complete response to chemotherapy, absence of recurrent disease, and lymphovascular space invasion were significant predictors of survival in patients with ovarian carcinoma. Herein, the present results are following the outcomes of the other authors. Given the fact that some authors have shown that some patients with complete response to chemotherapy did not have significantly higher survival even if the number of therapy cycles was higher than six. Of note, that indicates that efforts should be made to identify tumor characteristics that could determine patients who will benefit from the standard treatment and those who need other, tailored treatment, preventing overtreatment. Herewith, emerging data regarding precision medicine and molecular-based personalized treatment modalities are promising and will likely modify the way the authors provide multiple lines of treatments in the near future. *Bene diagnoscitur bene curatur.*

## Data availability

The clinical data used to support the findings of this study are available from the corresponding author upon request.

## Funding

None declared.

## CRediT authorship contribution statement

**Bojana Gutic:** Conceptualization, Data curation, Formal analysis, Investigation, Methodology, Project administration, Resources, Validation, Visualization, Writing – original draft. **Tatjana Bozanovic:** Investigation, Methodology, Project administration, Resources, Validation, Visualization. **Aljosa Mandic:** Investigation, Methodology, Project administration, Resources, Validation, Visualization. **Stefan Dugalic:** Investigation, Methodology, Project administration, Resources, Validation, Visualization. **Jovana Todorovic:** Investigation, Methodology, Project administration, Resources, Validation, Visualization. **Miroslava Gojnic Dugalic:** Investigation, Methodology, Project administration, Resources, Validation, Visualization. **Demet Sengul:** Investigation, Methodology, Resources, Software, Supervision, Validation, Visualization, Writing – original draft, Writing – review & editing. **Dzenana A. Detanac:** Investigation, Methodology, Validation, Visualization, Writing – review & editing. **Ilker Sengul:** Investigation, Methodology, Project administration, Resources, Software, Supervision, Validation, Visualization, Writing – original draft, Writing – review & editing. **Dzemail Detanac:** Investigation, Methodology, Validation, Visualization, Writing – review & editing. **Tugrul Kesicioglu:** Investigation, Methodology, Validation, Visualization. **José Maria Soares Junior:** Investigation, Methodology, Project administration, Software, Validation, Visualization, Writing – review & editing.

## Conflicts of interest

The authors declare no conflicts of interest.
